# Determinants for hospitalisations, intensive care unit admission and death among 20,293 reported COVID-19 cases in Portugal, March to April 2020

**DOI:** 10.2807/1560-7917.ES.2021.26.33.2001059

**Published:** 2021-08-19

**Authors:** Vasco Ricoca Peixoto, André Vieira, Pedro Aguiar, Paulo Sousa, Carlos Carvalho, Daniel Thomas, Alexandre Abrantes, Carla Nunes

**Affiliations:** 1 NOVA National School of Public Health, Public Health Research Centre, Universidade NOVA de Lisboa, Lisbon, Portugal; 2 European Programme for Intervention Epidemiology Training (EPIET), European Centre for Disease Prevention and Control (ECDC), Stockholm, Sweden; 3 Unit for Multidisciplinary Research in Biomedicine, Abel Salazar Institute of Biomedical Sciences, Universidade do Porto, Porto, Portugal; 4 Communicable Disease Surveillance Centre, Public Health Wales, Cardiff, United Kingdom; 5 Comprehensive Health Research Centre, Universidade Nova de Lisboa

**Keywords:** COVID-19, determinants, risk factors, hospital admission, intensive care, death

## Abstract

**Background:**

Determinants of hospitalisation, intensive care unit (ICU) admission and death are still unclear for COVID-19. Few studies have adjusted for confounding for different clinical outcomes including all reported cases within a country.

**Aim:**

We used routine surveillance data from Portugal to identify risk factors for severe COVID-19 outcomes, and to support risk stratification, public health interventions, and planning of healthcare resources.

**Methods:**

We conducted a retrospective cohort study including 20,293 laboratory-confirmed cases of COVID-19 reported between 1 March and 28 April 2020 through the national epidemiological surveillance system. We calculated absolute risk, relative risk (RR) and adjusted relative risk (aRR) to identify demographic and clinical factors associated with hospitalisation, ICU admission and death using Poisson regressions.

**Results:**

Increasing age (≥ 60 years) was the major determinant for all outcomes. Age ≥ 90 years was the strongest determinant of hospital admission (aRR: 6.1), and 70–79 years for ICU (aRR: 10.4). Comorbidities of cardiovascular, immunodeficiency, kidney and lung disease (aRR: 4.3, 2.8, 2.4, 2.0, respectively) had stronger associations with ICU admission, while for death they were kidney, cardiovascular and chronic neurological disease (aRR: 2.9, 2.6, 2.0).

**Conclusions:**

Older age was the strongest risk factor for all severe outcomes. These findings from the early stages of the COVID-19 pandemic support risk-stratified public health measures that should prioritise protecting older people. Epidemiological scenarios and clinical guidelines should consider this, even though under-ascertainment should also be considered.

## Introduction

Previous studies of clinical outcomes of coronavirus disease (COVID-19) in China [1], Italy [2,3] and the United States (US) [4] have described risk factors for poorer clinical outcomes, including age, sex and comorbidities without adjusting for confounding. Identifying these determinants and their isolated risk can help inform health policy on risk stratification and implementation of public health measures, but also improve epidemiological scenarios and forecasts on the needed healthcare resources.

Other studies on risk factors for clinical outcomes of COVID-19 have included small series of patients, mostly among those hospitalised with severe disease [5-7], making it difficult to produce reliable estimates for specific risk factors in the general population.

There is still uncertainty about the contribution of each factor for hospitalisation, intensive care unit (ICU) admission and death in the general population, as only a few studies conducted multivariable analysis to account for confounding [8-10]. One study of laboratory-confirmed COVID-19 cases in New York City, US, found an increased risk of hospitalisation for those aged 65 years and older when compared with those 19–44 years [8]. A large cross-sectional survey in the United Kingdom (UK) describing 16,749 patients hospitalised with COVID-19 showed a higher risk of death for patients with increasing age, cardiovascular, pulmonary or kidney disease, as well as malignancy, dementia and obesity [9]. The largest cohort study to date (as at May 2020), the OpenSAFELY Collaborative study [10], was conducted in the UK and included 17 million adult patients under the National Health Service (NHS). This study found that being a man, being older, and living in a more socioeconomically deprived community, as well as having uncontrolled diabetes, severe asthma, or other comorbidities were relevant risk factors for death by COVID-19.

High quality data on population level risk factors for poor outcomes of COVID-19 is needed to inform public health policy and preparedness. Compared with several other European countries, in March and April 2020, Portugal had a high estimated case ascertainment, ranging from 22% (95% confidence interval (CI): 18–39) [11] to 36.6% (95% CI: 29.5–45.7) [12], and it had a high testing rate in mid-May [13], despite having lower transmission levels, lower case fatality rate (CFR) and lower test positivity rates [14].

We aimed to further understand COVID-19 risk factors for three different outcomes – hospitalisation, ICU admission and death – in order to better support risk stratification, clinical and public health interventions and healthcare resource planning in Portugal.

## Methods

### Study design and data sources

We conducted a retrospective cohort study including all reported confirmed cases of COVID-19 in Portugal (n = 20,293) during the first 2 months of the pandemic (from 1 March to 28 April 2020). Outcomes measured were hospitalisation, ICU admission and death. We calculated absolute risk, relative risk (RR) and adjusted relative risk (aRR) for age, sex, comorbidities and region of occurrence using Poisson regression.

We obtained anonymised data from the Portuguese Directorate-General of Health (DGS), including all confirmed cases of COVID-19 notified to the national epidemiological surveillance system (Sistema Nacional de Vigilância Epidemiológica, SINAVE). SINAVEmed is an electronic platform that includes information about clinical findings and comorbidities; clinicians are obliged by law to notify all suspected and confirmed cases of COVID-19. Notifications trigger an epidemiological investigation by the local public health services, where a public health physician (health authority in the area of residence of the case) validates the case. At a later stage, the regional public health department and, finally, DGS conduct a final validation of case information. 

### Case definitions

A confirmed case of COVID-19 is defined as anyone with positive result for severe acute respiratory syndrome coronavirus 2 (SARS-CoV-2) RNA by RT-PCR in nasopharyngeal and/or oropharyngeal specimens regardless of clinical or epidemiological criteria. However, if these criteria were present, the likelihood of being tested and confirmed was higher.

Up until 26 March 2020, all patients with fever, cough or dyspnoea and contact with a symptomatic case or returning from an active transmission zone (outside Portugal) were considered suspect cases and had indication to be tested. From 26 March, all people with fever, onset of cough or dyspnoea, regardless of epidemic link, were considered suspect and told by public health officials to call the Portuguese NHS Health line and were subsequently sent for testing.

### Outcomes

We evaluated three primary outcomes: hospitalisation in the general ward (not ICU), admission to ICU and death. Outcomes were considered according to data from SINAVEmed, completed as described above. Outcome data are registered at the local level, but are updated retrospectively at the regional and national level (DGS).

### Risk factors

From the SINAVEmed dataset, we included the following variables: age, sex, chronic diseases/comorbidities (asthma, cancer, cardiovascular disease, diabetes (all types), immunodeficiencies (including HIV), kidney disease, liver disease, lung disease (other than asthma), haematological disease, chronic neurological disease (including dementia)) and region of occurrence of the case. Regions were included for adjustment.

### Statistical analysis

Descriptive statistics were applied to characterise the cohort of confirmed COVID-19 cases and the distribution by outcomes (Supplementary Material). We conducted univariable analysis and calculated absolute risks (proportion where each outcome was observed by stratum), RR with 95% CI and p values (Wald test). We then calculated aRR through multivariable analysis, using Poisson regression models that included the same co-variables for each outcome.

Age was divided into six categories with a reference group of 0–49 years and groups with increments of 10 years up to 90 years and above.

Variables with inconclusive or missing data were classified as follows: for the outcome ICU admission, ‘unknown’ was considered ‘no’; for death, ’still in treatment’ was considered ‘no’ and unknown data for death was not included in the analysis. Where available, these variables were filled as ‘yes’ retrospectively through cross-reference from other sources at the national level (real time national digital registry of deaths and hospital registries). Both of these outcomes agreed with aggregated data officially reported during the corresponding time period. Missing data for other non-mandatory variables like comorbidities were assumed ‘no’ because of the notification form architecture. For admissions to the hospital ward, missing outcomes were removed from the analysis. Finally, we built forest plots with aRR for the Poisson regression and CI for the three clinical outcomes analysed.

**Figure 1:**
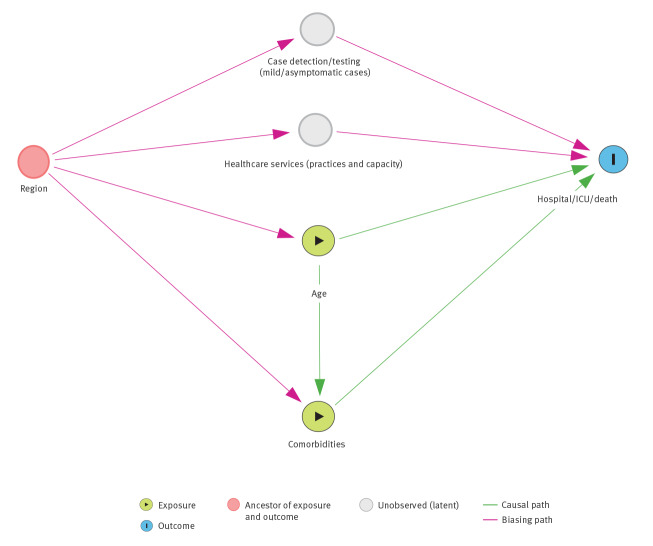
Illustration of assumptions included in the model for associations between exposures of interest and confounding variables

To make explicit the assumptions behind variables included in the models, we drew a directed acyclic graph [15] on the relations between variables and potentially biasing pathways considering exposures of interest and potential bias (Figure 1).

ICU: intensive care unit.

The directed acrylic graph was generated using the R package DAGggity [15].

The regression models were analysed in Stata (version 14, StataCorp, College Station, Texas, US). All analyses used 95% CI and considered a p value < 0.05 as statistically significant.

### Ethical statement

Data were shared by DGS with the National School of Public Health–NOVA University of Lisbon under a partnership for COVID-19 research. The Ethical Committee of the National School of Public Health approved the project (Approval: CE/ENSP/CREE/2/2020).

## Results

Of 20,293 laboratory-confirmed cases of COVID-19, 2,972 (14.6%) were admitted to hospital (general ward), 261 (1.3%) were admitted to the ICU and 502 (2.5%) died. Of the total cases, 41.3% were men, 58.7% were above 50 years of age and 17.0% had at least one recorded comorbidity. Despite having only approximately one third of the Portuguese population, the North region had 60% of all cases (Supplementary Material).

### Risk factors for hospitalisation

In the univariable analysis, absolute risk of hospitalisation increased with age, which was the strongest risk factor. Age groups above 60 years of age presented an aRR of hospitalisation higher than any chronic disease after adjustment, assuming 0–49 years of age as a reference.

Different regions had varying hospitalisation risks and statistically significant differences in RR that were maintained after full adjustment. Comorbidities with higher aRR were immunodeficiencies (aRR: 1.8; 95% CI: 1.4–2.3), cardiovascular disease (aRR: 1.8; 95% CI: 1.5–2.2), kidney disease (aRR: 1.6; 95% CI: 1.4–1.7), liver disease (aRR: 1.5; 95% CI: 1.2–2.0 and neurological disease (aRR 1.8; 95% CI 1.7–2.0) (Table 1).

**Table 1:** Association between geodemographic factors, comorbidities and hospitalisations among COVID-19 cases, Portugal, 1 March–28 April 2020 (n = 18,670^a^)

Characteristics	Total (n)	Hospitalisations^b^ (n)	Hospitalisations^b^ (%)	Crude RR	95% CI	p value	aRR	95% CI	p value
Sex									
Women	10,949	1,416	12.9	Ref.					
Men	7,721	1,556	20.2	1.6	1.5–1.7	< 0.001	1.4	1.4–1.5	< 0.001
Age (years)									
0–49	9,055	462	5.1	Ref.					
50–59	3,325	336	10.1	2.0	1.7–2.3	< 0.001	1.9	1.7–2.2	< 0.001
60–69	2,233	491	22.0	4.3	3.8–4.9	< 0.001	3.7	3.3–4.1	< 0.001
70–79	1,653	659	39.9	7.8	7.0–8.7	< 0.001	5.7	5.1–6.4	< 0.001
80–89	1,678	747	44.5	8.7	7.9–9.7	< 0.001	6.4	5.8–7.2	< 0.001
≥ 90	726	277	38.2	7.5	6.6–8.6	< 0.001	6.1	5.4–7-0	< 0.001
Region^c^									
North	11,090	1,453	13.1	Ref.					
Acores	48	14	29.2	2.2	1.4–3.5	0.001	2.7	1.8–4.2	< 0.001
Alentejo	370	54	14.6	1.1	0.9–1.4	0.403	1.3	1.0–1.6	0.033
Algarve	462	92	19.9	1.5	1.3–1.8	< 0.001	1.7	1.4–2.0	< 0.001
Center	2,651	510	19.2	1.5	1.3–1.6	< 0.001	1.2	1.1–1.3	< 0.001
Lisbon and Tagus Valley	3,951	827	20.9	1.6	1.5–1.7	< 0.001	1.6	1.5–1.7	< 0.001
Madeira	87	15	17.2	1.3	0.8–2.1	0.255	2.0	1.4–3.0	0.001
Comorbidities									
Asthma	258	26	10.1	0.6	0.4–0.9	0.01	0.9	0.6–1.2	0.356
Cancer	579	292	50.4	3.4	3.1–3.7	< 0.001	1.4	1.3–1.6	< 0.001
Cardiovascular disease	52	49	94.2	6.0	5.6–6.5	< 0.001	1.8	1.5–2.2	< 0.001
Diabetes	1,057	496	46.9	3.3	3.1–3.6	< 0.001	1.4	1.3–1.5	< 0.001
Immunodeficiencies	99	43	43.4	2.8	2.1–3.5	< 0.001	1.8	1.4–2.3	< 0.001
Kidney disease	382	273	71.5	4.8	4.5–5.2	< 0.001	1.6	1.4–1.7	< 0.001
Liver disease	102	64	62.7	4.0	3.4–4.7	< 0.001	1.5	1.2–2.0	< 0.001
Lung disease	637	292	45.8	3.1	2.8–3.4	< 0.001	1.4	1.3–1.5	< 0.001
Haematological disease	202	131	64.9	4.2	3.8–4.7	< 0.001	1.4	1.2–1.6	< 0.001
Neurological disease	733	476	64.9	4.7	4.4–5.0	< 0.001	1.8	1.7–2.0	< 0.001

aRR: adjusted risk reduction; CI: confidence interval; COVID-19: coronavirus disease; Ref.: reference; RR: risk reduction.

^a ^Only 18,670 people included in the study had a known hospital admission status.

^b ^Hospitalisations refer to those in the general ward.

^c ^The total for the regions was 18,659 cases; 11 cases were missing.

The area under the ROC curve (AUC) was 0.823 (asymptotic 95% CI: 0.814–0.831).

### Risk factors for intensive care unit admission

There was a consistent increase in risk of admission to the ICU with increasing age up to 70–79 years (aRR: 10.4; 95% CI: 6.5–16.6, but reduced in the subsequent age groups. These findings were maintained after adjustment. As observed for hospitalisation, different regions had varying ICU risks and statistically significant differences in RR that were maintained after adjustment. Among regions with cases in the ICU, the North region had the lowest risk. The diseases with higher aRR for admission to the ICU were cardiovascular disease (aRR: 4.3; 95% CI: 2.5–7.4, immunodeficiencies (aRR: 2.8; 95% CI: 1.3–5.7), kidney disease (aRR: 2.4; 95% CI: 1.6–3.7), and lung disease (aRR: 2.0; 95% CI: 1.4–2.9). Liver disease and neurological disease were associated with hospitalisation but not with ICU admission. Age groups above 50 years had higher aRR than any chronic disease alone. The adjusted risk of admission to the ICU in cases aged 70–79 years was more than 10 times the risk of cases aged 0–49 years but aRR reduced after 79. Regions maintained small, but statistically significant differences after adjustment (Table 2).

**Table 2:** Association between geodemographic factors, comorbidities and intensive care unit admission among COVID-19 cases, Portugal, 1 March–28 April 2020 (n = 20,293)

Characteristics	Total (n)	ICU (n)	ICU (%)	Crude RR	95% CI	p value	aRR	95% CI	p value
Sex									
Women	11,903	88	0.7	Ref.					
Men	8,390	173	2.1	2.8	2.2–3.6	< 0.001	2.2	1.7–2.9	< 0.001
Age (years)									
0–49	9,675	25	0.3	Ref.					
50–59	3,549	40	1.1	4.4	2.7–7.2	0.005	4.5	2.8–7.3	< 0.001
60–69	2,463	67	2.7	10.5	6.7–16.6	< 0.001	8.8	5.6–13.7	< 0.001
70–79	1,808	70	3.9	15.0	9.5–23.6	< 0.001	10.4	6.5–16.6	< 0.001
80–89	1,932	50	2.6	10.0	6.2–16.1	< 0.001	7.3	4.4–12.1	< 0.001
≥ 90	866	9	1.0	4.0	1.9–8.6	0.01	3.8	1.8–8.2	0.001
Region									
North	12,207	101	0.8	Ref.					
Acores	48	3	6.3	7.6	2.5–23.0	< 0.001	9.3	4.0–22.0	< 0.001
Alentejo	387	9	2.3	2.8	1.4–5.5	0.002	3.2	1.6–6.3	0.001
Algarve	472	18	3.8	4.6	2.8–7.6	< 0.001	5.2	3.2–8.2	< 0.001
Center	2,812	44	1.6	1.9	1.3–2.7	< 0.001	1.8	1.3–2.5	0.001
Lisbon and Tagus Valley	4,264	85	2.0	2.4	1.8–3.2	< 0.001	2.5	1.9–3.3	< 0.001
Madeira	90	0	0.0	0.0	NA	0.386	0.0	0.0–0.0	< 0.001
Comorbidities									
Asthma	277	4	1.4	1.1	0.4–3.0	0.814	1.6	0.6–4.4	0.334
Cancer	611	22	3.6	3.0	1.9–4.6	< 0.001	1.1	0.7–1.8	0.549
Cardiovascular disease	54	10	18.5	14.9	8.4–26.5	< 0.001	4.3	2.5–7.4	< 0.001
Diabetes	1,145	53	4.6	4.3	3.1–5.7	< 0.001	1.7	1.3–2.3	0.001
Immunodeficiencies	107	7	6.5	5.2	2.5–10.8	< 0.001	2.8	1.3–5.7	0.006
Kidney disease	400	34	8.5	7.5	5.3–10.5	< 0.001	2.4	1.6–3.7	< 0.001
Liver disease	107	5	4.7	3.7	1.6–8.8	0.002	0.6	0.3–1.7	0.361
Lung disease	688	37	5.4	4.7	3.4–6.6	< 0.001	2.0	1.4–2.9	< 0.001
Haematotological disease	221	8	3.6	2.9	1.4–5.7	0.002	1.1	0.5–2.3	0.823
Neurological disease	794	25	3.2	2.6	1.7–3.9	< 0.001	1.1	0.7–1.7	0.668

aRR: adjusted risk reduction; CI: confidence interval; COVID-19: coronavirus disease; ICU: intensive care unit; NA: not applicable; Ref.: reference; RR: risk reduction.

The area under the ROC curve (AUC) was 0.833 (asymptotic 95% CI: 0.811–0.854).

**Figure 2:**
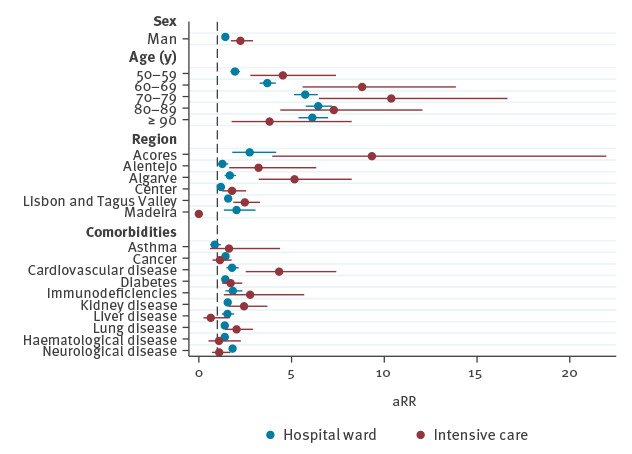
Adjusted risk for hospital (n = 18,670) and intensive care unit (n = 20,293) admission among COVID-19 cases using a Poisson regression model, Portugal, 1 March–28 April 2020

A forest plot with a visual representation of aRR and 95% CI for hospital ward admission and ICU admission can be seen in Figure 2.

COVID-19: coronavirus disease.

Reference categories are sex: woman; age: 0–49 years; region: North.

### Risk factors for death

Risk of death was disproportionally affected by age. We observed that there was a constant increase of risk of death with age, unlike what was seen with hospitalisation and ICU admissions. Among cases aged 0–49 years, the CFR was 0.04%, contrasting with a CFR of 12.9% among those older than 90 years of age. The aRR increased more significantly from the age group 70–79 years (aRR: 112.7; 95% CI: 41.2–308.5) and older. Different regions had slightly different CFRs and statistically significant differences of the aRR for three regions. The comorbidities with higher aRR were kidney (aRR: 2.9; 95% CI: 2.3–3.7), cardiovascular (aRR: 2.6; 95% CI: 1.7–3.9), and neurological disease (aRR: 2.0; 95% CI: 1.7–2.5) (Table 3).

**Table 3:** Association between geodemographic factors, comorbidities and death among COVID-19 cases, Portugal, 1 March–28 April 2020 (n = 20,270^a^)

Characteristics	Total (n)	Deaths (n)	CFR (%)	Crude RR	95% CI	p value	aRR	95% CI	p value
Sex									
Women	11,900	253	2.1	Ref.					
Men	8,370	249	3.0	1.4	1.2–1.7	0	1.4	1.189–1.68	< 0.001
Age (years)									
0–49	9,675	4	0.0	Ref.					
50–59	3,548	15	0.4	10.2	3.4–30.8	< 0.001	9.8	3.3–29.6	< 0.001
60–69	2,459	44	1.8	43.3	15.6–120.3	< 0.001	37.1	13.3–103.4	< 0.001
70–79	1,800	116	6.4	155.9	57.6–421.8	< 0.001	112.7	41.2–308.5	< 0.001
80–89	1,924	212	11.0	266.5	99.2–715.8	< 0.001	179.1	65.6–489.0	< 0.001
≥ 90	864	111	12.9	310.7	114.9–840.5	< 0.001	226.8	82.7–622.1	< 0.001
Region^b^									
North	12,196	315	2.6	Ref.					
Acores	48	0	0.0	0.0	NA	0.259	0.0	0.0–0.0	< 0.001
Alentejo	387	6	1.6	0.6	0.3–1.3	0.205	0.8	0.4–1.8	0.578
Algarve	470	6	1.3	0.5	0.2–1.1	0.077	0.6	0.3–1.4	0.268
Center	2,805	93	3.3	1.3	1.0–1.6	0.031	0.9	0.7–1.1	0.352
Lisbon and Tagus Valley	4,261	74	1.7	0.7	0.5–0.9	0.002	0.7	0.5–0.8	0.001
Madeira	90	0	0.0	0.0	NA	0.122	0.0	0.0–0.0	< 0.001
Comorbidities									
Asthma	277	3	1.1	0.4	0.1–1.3	0.133	0.7	0.2–2.2	0.581
Cancer	603	47	7.8	3.4	2.5–4.5	< 0.001	1.3	0.9–1.7	0.151
Cardiovascular disease	53	19	35.8	15.0	10.4–21.7	< 0.001	2.6	1.7–3.9	< 0.001
Diabetes	1,144	83	7.3	3.3	2.6–4.16	< 0.001	1.0	0.8–1.3	0.985
Immunodeficiencies	107	6	5.6	2.3	1.0–5.0	0.037	1.5	0.7–3.3	0.295
Kidney disease	400	98	24.5	12.1	9.9–14.7	< 0.001	2.9	2.3–3.7	< 0.001
Liver disease	107	7	6.5	2.7	1.3–5.5	0.007	0.8	0.4–2.0	0.687
Lung disease	686	60	8.7	3.9	3.0–5.0	< 0.001	1.3	1.0–1.7	0.062
Haematological disease	220	29	13.2	5.6	3.9–7.9	< 0.001	1.2	0.8–1.8	0.3
Neurological disease	790	123	15.6	8.0	6.6–9.7	< 0.001	2.0	1.7–2.5	< 0.001

aRR: adjusted risk reduction; CFR: case fatality rate; CI: confidence interval; COVID-19: coronavirus disease; Ref.: reference; RR: risk reduction.

The area under the ROC curve (AUC) was 0.909 (asymptotic 95% CI: 0.900–0.918).

^a^ Data was missing for outcome of death for 23 cases.

^b^ The total for the regions was 20,257 cases; 13 cases were missing.

**Figure 3:**
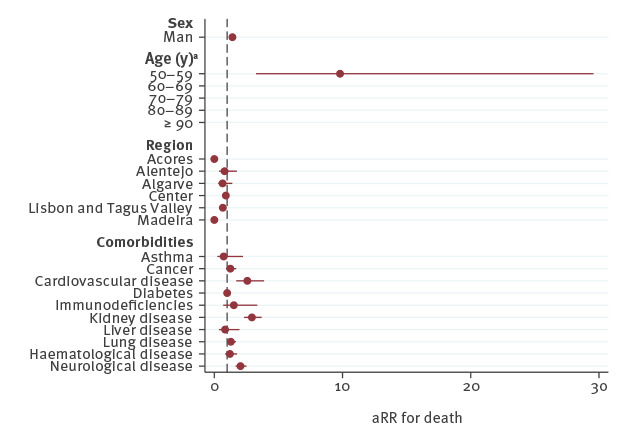
Adjusted risk reduction for death among COVID-19 cases using a Poisson regression model, Portugal, 1 March–28 April 2020 (n = 20,270)

A forest plot representing the aRR and 95% CI can be seen below in Figure 3.

COVID-19: coronavirus disease.

Reference categories are sex: woman; age: 0–49 years; region: North.

^a^Age Risk: 50–59 years (aRR: 9.8; 95% CI: 3.3–29.6); 60–69 years (aRR: 37.1; 95% CI: 13.3–103.4); 70–79 years (aRR: 112.7; 95% CI: 41.2–308.5); 80–89 years (aRR: 9179.1; 95% CI: 65.6–489.0); ≥ 90 years (aRR: 226.8; 95% CI: 82.7–622.1).

## Discussion

This study performed during the early stage of the COVID-19 pandemic, was designed to identify which demographic and clinical factors were associated with severe acute outcomes of COVID-19 in the Portuguese population, specifically hospitalisation, ICU admission and death. We used data from all reported cases during the first 2 months of the epidemic in Portugal and have presented RR adjusted for confounding.

We used national epidemiological information extracted from the SINAVEmed electronic platform. This information was validated by the public health authorities network in Portugal at the local, regional and national level, which contributed to data quality and allowed better population risk estimates in that period. Portugal had, at the time, one of the highest testing rates per capita, one of the lowest CFR and a low test positivity rate in Europe [13,14]. Estimates by the London School of Hygiene and Tropical Medicine and by the Imperial College London estimated one of the lowest under-reporting/under-ascertainment in Portugal [11,12]. However, varying levels of under-ascertainment in different age groups could lead to bias in risk estimates. If younger age groups were less likely to be diagnosed because they may have milder symptoms or symptoms that are not included in testing guidelines, our risk estimates could be under-estimated for older age groups. It is probable that the overall sensitivity of the surveillance system was lower before 26 March 2020, as the testing strategy was less broad and only symptomatic cases with an epidemic link were tested. Some regional differences in testing strategies are probably relevant, hence we adjusted for this possible confounding. 

In this study, we found that increasing age was, as in other multivariable analysis [8,10], the most relevant risk factor for hospitalisation, ICU admission and death [10]. The very high values of aRR for older ages (≥ 60 years) in comparison with other risk factors can be explained by the very low case fatality observed in the reference age group 0–49 years irrespective of comorbidities, which are less frequent in this group. There is potentially a higher under-estimation in men, as with other diseases where health-seeking behaviour differs, namely for respiratory symptoms [16]. This, in turn, might have led to an overestimation of the risk. However, other biological differences may explain part of the increased risk in men.

Hospitalisation and ICU admissions had a relevant increase in risk in age groups 60–69 and 70–79 years. The risk of ICU admission was reduced after 70–79 years. This is not expected to be due to negative selection based on age since ICU bed occupation did not exceed 60% during that period [17] and there are guidelines with criteria for ICU admission in Portugal [18] that consider only clinical severity. Other studies found similar situations for influenza [19,20]. It is possible that some older patients may die without meeting criteria for ICU admission. Also, patients who meet those criteria may either die before they can be admitted, or are not admitted because they have no expected clinical benefit and very low recovery expectations. However, further research is needed to understand the benefit of ICU treatment among very elderly patients. Debate has been ongoing on this topic considering the challenges and ethics of admitting very elderly patients to the ICU, respecting patient and family wishes and therapeutic futility [21-25].

Older age was by far the most important determinant for COVID-19-associated death. Most comorbidities were associated with increased risk for hospitalisation, ICU admission and death, especially cardiovascular, kidney, respiratory and neurological disease, although risk varied for different outcomes. All comorbidities increased risk more homogenously for hospitalisation than for ICU admission and death. This could be explained by lower thresholds for the decision to admit patients to a general ward vs ICU, but also by Portuguese guidelines that consider the existence of comorbidities for hospital admission but include mainly clinical severity criteria for ICU [18]. Risk factors for death vary from other outcomes possibly because death does not include a clinical management decision. For ICU admission, the most relevant risk factors besides age were cardiovascular disease, immunodeficiencies, kidney disease, and lung disease, while liver, neurological and haematological disease were not significantly associated.

Asthma was not a risk factor for any outcome, in line with what was found in other studies [8,9]. However, the largest cohort investigated to date found a slight increase in adjusted risk of death for asthma in severe cases [10].

We found particularly strong associations of older age, cardiovascular disease and chronic kidney disease for both ICU admission (60–79 years) and death (≥ 60) even though risk for ICU reduced in those above 80 years. A weaker association with chronic lung disease was observed, as in a similar study [8], although the largest cohort study examining the outcome of death with COVID-19 found that lung disease had the highest risk among comorbidities [10].

For the outcome of death, our results were similar to the International Severe Acute Respiratory and Emerging Infection Consortium (ISARIC) study [9] and to the OpenSafely Project Cohort [10], although the differences in risk measures for age were larger. Kidney, cardiovascular and chronic neurological disease were the comorbidities with a stronger association with death. Although not specified, most neurological disease is expected to be dementia, considering the epidemiology of neurological diseases [26].

Regions were included in the models primarily to minimise potential confounding. We found small differences in the association of region with outcomes after adjustment. Since some heterogeneity naturally exists between regional epidemic situations, testing strategies and case ascertainment, we hypothesised that some of the differences observed between regions were primarily due to different testing and case ascertainment, but possibly due to admission practices and different treatment quality or access [8]. Significant differences in healthcare service response were not expected since guidelines with criteria for admission to general wards and the ICU have been issued by the DGS [18] and capacity was not breached. It is not clear if case ascertainment may have been higher in the North region. Most cases were reported from this region and, while the chance of admission to the ICU was lower, the risk of death was higher.

The study has limitations. The extracted data are routine surveillance data from an early phase of the epidemic in Portugal and as such, estimated risks might change with increased testing, broadened testing criteria that included a larger spectrum of symptoms, higher detection of mild and asymptomatic infection, changes in regional incidence and hospital healthcare care demand and further data validation. There are also other relevant comorbidities that we could not adjust for that have been previously found to be of relevance for the COVID-19 severity outcomes, such as obesity [8-10], economic deprivation [10] and minority ethnic groups [10,27]. Hypertension is also not included since it was not available in SINAVEmed dataset, although recent evidence from the largest cohort to date suggest that controlled hypertension alone does not increase the risk of death of COVID-19 patients [10]. In our study, no data on smoking were available but, as with hypertension, it does not seem to be a relevant factor for poorer outcomes [10].

The small numbers observed with cardiovascular disease are because, contrary to other comorbidities, cardiovascular disease is not a specific variable in the SINAVEmed notification form. As such, physicians must specifically write that condition in the field ‘other chronic conditions’ on the form. This may introduce information bias as the field ‘other chronic conditions’ may be more frequently completed for cases with poorer outcomes, overestimating the risk for cardiovascular disease. However, other studies conducting multivariable analysis from cases have found cardiovascular disease to be one of the most relevant risk factors [8-10]. People with this and other comorbidities such as asthma may also have been more compliant with prevention measures.

It is possible that some cases in the dataset could have been admitted to the hospital or died after the collected data was made available for research purposes. We believe this would not introduce a systematic error in risk estimates since this situation is expected to be relatively rare considering the large sample size. We found no strong reason for those cases to be significantly different from those where the outcome had already been reported and numbers were coherent with officially reported data during that period.

We used aRR in multivariable analysis using Poisson regressions assuming constant time of exposure. The use of an adjusted odds ratio to estimate an aRR can be appropriate for studies of rare outcomes (< 10%) but may overestimate the risk if outcomes are more frequent [28-30]. Overestimating RR could inappropriately affect clinical decision making, policy development and priority setting, as well as economic evaluation and targeted prevention programmes or treatments [28,29]. Poisson regression is likely to compute confidence intervals that are conservative with more common outcomes.

We categorised age using 0–49 years as reference since other studies conducting multivariable analysis have used such age categorisation [9], including the largest cohort studied for risk factors for death from COVID-19 [10]. Low risk was found in this group for acute severe outcomes [8-10].

Our findings aimed to help shape public health policy by modelling risk criteria, thereby aiding in prediction of healthcare needs in the face of different epidemic forecasts. Clinical risk assessment tools could be built to aid clinical decisions related to admission to the hospital or ICU, since most patients can be safely followed up at home [31]. Policy recommendations and public health intervention like vaccination in Portugal and other European countries may consider specific comorbidities and age cut-offs when defining people at risk. As such, it is relevant to understand what level of risk is added by each specific characteristic. In Portugal, patients with certain comorbidities may be granted medically justified absences from work if they cannot work from home [32] and have been prioritised for vaccination. Our findings reinforce that if these patients become ill with COVID-19, they will be at increased risk of hospitalisation, ICU admission and/or death.

Considering absolute risk estimates of specific outcomes among COVID-19-infected people in different age groups or individual risk estimates using models parameters, researchers must consider under-ascertainment of mildly symptomatic and asymptomatic cases [33]. However, for RR estimates, differential under-ascertainment is necessary to introduce bias. Under-ascertainment is probably higher in younger ages [34] and lower with older age as older people may have a lower threshold for testing and have a smaller proportion of mild and asymptomatic or paucisymptomatic infections. This could mean modelled RR estimates were underestimated for older patients while absolute risk for younger patients was overestimated. It will be relevant to revaluate the same data at a later time point to examine whether the findings are maintained and to generate hypotheses on the reasons behind those changes.

This work was one of the references used by the Portuguese authorities for initial selection of priority groups for vaccination, considering age and combinations of age and relevant comorbidities, with the aim of preventing COVID-19-related hospital admissions and deaths in the face of initially limited vaccine availability [35]. Our study adds evidence supporting the importance of effective measures specifically targeted at protecting older population [36].

### Conclusions

Age should be considered as the strongest single risk factor for all measured COVID-19 outcomes. This finding should be taken into account both in terms of prevention strategies (e.g. public health measures, vaccination priorities, healthcare demand scenarios) and in terms of clinical management and prognosis. Comorbidities also have an impact on clinical outcomes (especially cardiovascular, kidney, lung disease, immunodeficiencies and neurological disease) but the associations were weaker than age and varied for different outcomes.

RR had larger increases after 60 years of age for the outcome death, as CFR was low in those aged 49 years and under. Comprehensive epidemiological surveillance of settings with a high risk population, including long-term care facilities, and prevention and control measures to protect the older population and those around them are part of an efficient strategy to reduce hospital admission and deaths. In addition, a strong and innovative communication plan targeted to the public to protect older people should be pursued.

Risk-stratified public health measures should primarily consider age but individual preventive behaviours should be promoted across all age groups to reduce overall spread and ultimately prevent infection in higher risk groups. However, strategies aimed at protecting ‘only’ those at higher risk might end up being inefficient, impractical or unethical [37]. Moreover, other negative long-term effects of the disease on health among lower risk groups are still uncertain and could have a relevant future impact [38]. These include COVID-19-associated syndromes and conditions.

## Supplementary Data

731000 Supplement
