# Supplementary material for: Determinants for hospitalisations, intensive care unit admission and death among 20,293 reported COVID-19 cases in Portugal, March to April 2020
Source: Euro Surveill. 2021 Aug 19;26(33):2001059. doi: 10.2807/1560-7917.ES.2021.26.33.2001059 (PMC8380973; doi:10.2807/1560-7917.ES.2021.26.33.2001059)
Supplement: Supplement [file 20-01059_PEIXOTO_Supplement.pdf]

This supplementary material is hosted by Eurosurveillance as supporting information alongside the article [Determinants for hospitalisations, intensive care unit admission and death among 20,293 reported COVID-19 cases in Portugal, 1 March to 28 April 2020] on behalf of the authors who remain responsible for the accuracy and appropriateness of the content. The same standards for ethics, copyright, attributions and permissions as for the article apply. Supplements are not edited by Eurosurveillance and the journal is not responsible for the maintenance of any links or email addresses provided therein.

Appendix A. Supplementary Data

Table 1. Cohort characteristics, distribution of cases by strata in total and by outcome and chi-square p-value for distribution in different categories of each outcomes

	**Total *n* (%)**	**By Hospitalization *n* (%)**		*p*	**By Intensive Care *n* (%)**		*p*	**By Outcome *n* (%)**		*p*
	(N = 20293)	Yes	No or Unk		Yes	No or Unk		Death	Alive or In Treat.	
Age										
0-9	316 (1.6%)	18 (0.6%)	298 (1.7%)		0 (0.0%)	316 (1.6%)		0 (0.0%)	316 (316%)	
10-19	575 (2.8%)	24 (0.8%)	551 (3.2%)		1 (0.4%)	574 (2.9%)		0 (0.0%)	575 (2.9%)	
20-29	2297 (11.3%)	61 (2.1%)	2236 (12.9%)		4 (1.5%)	2293 (11.4%)		0 (0.0%)	2297 (11.6%)	
30-39	2957 (14.6%)	145 (4.9%)	2812 (16.2%)		5 (1.9%)	2952 (14.7%)		0 (0.0%)	2957 (14.9%)	
40-49	3530 (17.4%)	214 (7.2%)	3316 (19.1%)	<0.001	15 (5.7%)	3515 (17.5%)	<0.001	4 (0.8%)	3526 (17.8%)	<0.001
50-59	3549 (17.5%)	336 (11.3%)	3213 (18.5%)		40 (15.3%)	3509 (17.5%)		15 (3.0%)	3534 (17.9%)	
60-69	2463 (12.1%)	491 (16.5%)	1972 (11.4%)		67 (25.7%)	2396 (12.0%)		44 (8.8%)	2419 (12.2%)	
70-79	1808 (8.9%)	659 (22.2%)	1149 (6.6%)		70 (26.8%)	1738 (8.7%)		116 (23.1%)	1692 (8.5%)	
80-89	1932 (9.5%)	747 (25.1%)	1185 (6.8%)		50 (19.2%)	1882 (9.4%)		212 (42.2%)	1720 (8.7%)	
>90	866 (4.3%)	277 (9.3%)	589 (3.4%)		9 (3.4%)	857 (4.3%)		111 (21.1%)	755 (3.8%)	
Gender										
Female	11903 (58.7%)	1416 (47.6%)	10487 (60.5%)	<0.001	88 (33.7%)	11815 (59.0%)	<0.001	253 (50.4%)	11650 (58.9%)	<0.001
Male	8390 (41.3%)	1556 (52.4%)	6834 (39.5%)		173 (66.3%)	8217 (41.0%)		249 (49.6 %)	8141 (41.1 %)	
Region										
Norte	12207 (60.2%)	1453 (48.9%)	10754 (62.1%)		101 (38.7%)	12106 (60.4%)		315 (62.7%)	11892 (60.1%)	
Centro	2812 (13.9%)	510 (17.2%)	2302 (13.3%)		44 (16.9%)	2768 (13.8%)		93 (18.5%)	2719 (13.7%)	
LVT	4264 (21.0%)	827 (27.8%)	3437 (19.8%)	<0.001	85 (32.6%)	4179 (20.9%)	<0.001	74 (14.7%)	4190 (21.2%)	<0.001
Alentejo	387 (1.9%)	54 (1.8%)	333 (1.9%)		9 (3.4%)	378 (1.9%)		6 (1.2%)	381 (1.9%)	
Algarve	472 (2.3%)	92 (3.1%)	380 (2.2%)		18 (6.9%)	454 (2.3%)		6 (1.2%)	466 (2.4%)	

Madeira	90 (0.4%)	15 (0.5%)	75 (0.4%)		0 (0.0%)	90 (0.4%)		0 (0.0%)	90 (0.5%)	
Açores	48 (0,2%)	14 (0.5%)	34 (0.2%)		3 (1.1%)	45 (0.2%)		0 (0.0%)	48 (0.2%)	
Precondition										
Yes	3363 (16.6%)	1440 (48.5%)	1922 (11.1%)	<0.001	140 (53.6%)	3222 (16.1%)	<0.001	140 (53.6%)	3222 (16.1%)	<0.001
No or Unknown	16931 (83.4%)	1532 (51.5%)	15399 (88.9%)		121 (46.4%)	16810 (83.9%)		121 (46.4%)	16810 (83.9%)	
Specific Comorbidity										
Asthma	277 (1.4%)	26 (0.9%)	251 (1.4%)	0.013	4 (1.5%)	273 (1.5%)	0.814[a]	3 (0.6%)	274 (1.4%)	0.133
No Asthma	-	2946 (99.1%)	17070 (98.6%)		257 (98.5%)	19759 (98.6%)		499 (99.4%)	19517 (98.6%)	
Cancer	611 (3.0%)	292 (9.8%)	319 (1.8%)	<0.001	22 (8.4%)	589 (2.9%)	<0.001	47 (9.4%)	564 (2.8%)	<0.001
No Cancer	-	2680 (90.2%)	17002 (98.2%)		239 (91.6%)	19443 (97.1%)		455 (90.6%)	19227 (97.2%)	
Cardiac Disease	54 (0.3%)	49 (1.6%)	5 (<0.1%)	<0.001	10 (3.8%)	44 (0.2%)	<0.001[a]	19 (3.8%)	35 (0.2%)	<0.001[a]
No Cardiac Disease	-	2923 (96.4%)	17316 (99.9%)		251 (96.2%)	19988 (99.8%)		483 (96.2%)	19756 (99.8%)	
Diabetes	1145 (5.6%)	496 (16,7%)	649 (3.7%)	<0.001	53 (20.3%)	1092 (5.5%)	<0.001	83 (16.5%)	1062 (5.4%)	<0.001[a]
No Diabetes	-	2476 (83,3%)	16672 (96.3%)		208 (79.7%)	18940 (94.5%)		419 (83.5%)	18729 (94.6%)	
HIV	107 (0.5%)	43 (1.4%)	64 (0.4%)	<0.001	34 (13.0%)	100 (0.5%)	<0.001[a]	6 (1.2%)	101 (0.5%)	0.05
No HIV	-	2929 (98.6%)	17257 (99.6%)		227 (87%)	19932 (99.5%)		496 (98.8%)	19690 (99.5%)	
Kidney Disease	400 (2.0%)	273 (9.2%)	127 (0.7%)	<0.001	7 (2.7%)	366 (1.8%)	<0.001	98 (19.5%)	302 (1.5%)	<0.001
No Kidney Disease	-	2699 (90.8%)	17194 (99.3%)		254 (97.3%)	19666 (98.2%)		404 (80.5%)	19489 (98.5%)	
Liver Disease	107 (0.5%)	64 (2.2%)	43 (0.2%)	<0.001	5 (1.9%)	102 (0.5%)	0.008[a]	7 (1.4%)	100 (0.5%)	0.017[a]
No Liver Disease	-	2908 (97.8%)	17278 (99.8%)		256 (98.1%)	19930 (99.5%)		495 (98.6%)	19691 (99.5%)	
Lung Disease	688 (3.4%)	292 (9.8%)	396 (2.3%)	<0.001	37 (14.2%)	651 (3.2%)	<0.001	60 (12%)	628 (3.2%)	<0.001
No Lung Disease	-	2680 (90.2%)	16925 (97.7%)		224 (85.8%)	19381 (96.8%)		442 (88%)	19163 (96.8%)	
Hemato Disease	221 (1.1%)	131 (4.4%)	90 (0.5%)	<0.001	8 (3.1%)	213 (1.1%)	0.012[a]	29 (5.8%)	192 (1.0%)	<0.001
No Hemato Disease	-	2841 (95.6%)	17231 (99.5%)		253 (96.9%)	19819 (98.9%)		473 (94.2%)	19599 (99.0%)	
Neurologic Disease	794 (3.9%)	476 (16,0%)	318 (1,8%)	<0.001	25 (9.6%)	769 (3.8%)	<0.001	123 (24.5%)	671 (3.4%)	<0.001
No Neurologic Disease	-	2496 (84.0%)	17003 (98,2%)		236 (90.4%)	19263 (96.2%)		379 (35.5%)	19120 (96.6%)	
Pregnancy	119 (0.6%)	42 (1.4%)	77 (0.4%)	<0.001	0 (0.0%)	119 (0.6%)	0.411[a]	0 (0.0%)	119 (0.6%)	0.126[a]
No Pregnancy	-	2930 (98.6%)	17244 (99.6%)		261 (100.0%)	19913 (99.4%)		502 (100.0%)	19672 (99.4%)	
Hospitalization										
Yes	2972 (14.6%)	-	-	-	-	-	-	-	-	-
No or Unknown	17384 (85.4%)									

Intensive Care<br>Yes<br>Unknown	 261 (1.3%)<br>20032 (98.7%)	-	-	-	-	-	-	-	-	-
Respiratory Support<br>Yes<br>No or Unknown	 26 (0.1%)<br>20267 (99.9%)	-	-	-	-	-	-	-	-	-
Outcome<br>Alive<br>Death<br>Still in Treatment	 1244 (6.1%)<br>502 (2.5%)<br>18524 (91.3%)	-	-	-	-	-	-	-	-	
